# IMI-Type Carbapenemase-Producing *Enterobacter cloacae* Complex, France and Overseas Regions, 2012–2022

**DOI:** 10.3201/eid3006.231525

**Published:** 2024-06

**Authors:** Cécile Emeraud, Delphine Girlich, Manon Deschamps, Inès Rezzoug, Aymeric Jacquemin, Agnès B. Jousset, Solène Lecolant, Lucy Locher, Aurélien Birer, Thierry Naas, Rémy A. Bonnin, Laurent Dortet

**Affiliations:** INSERM, Université Paris-Saclay, Le Kremlin-Bicêtre, France (C. Emeraud, D. Girlich^,^ M. Deschamps, I. Rezzoug, A. Jacquemin, A.B. Jousset, T. Naas, R.A. Bonnin, L. Dortet);; Associated French National Reference Center for Antibiotic Resistance: Carbapenemase-Producing Enterobacteriaceae, Le Kremlin-Bicêtre (C. Emeraud, D. Girlich, I. Rezzoug, A.B. Jousset, T. Naas, R.A. Bonnin, L. Dortet);; Bicêtre Hospital, Assistance Publique-Hôpitaux de Paris, Le Kremlin-Bicêtre (C. Emeraud, I. Rezzoug, A.B. Jousset, S. Lecolant, L. Locher, T. Naas, L. Dortet);; Microbes, Intestin, Inflammation et Susceptibilité de l'Hôte (M2iSH), INRAE (Institut national de recherche pour l’agriculture, l’alimentation et l’environnement), Clermont-Ferrand, France (A. Birer);; Associated French National Reference Center for Antibiotic Resistance, CHU Gabriel-Montpied, Clermont-Ferrand (A. Birer)

**Keywords:** Enterobacter cloacae, carbapenemase, bacteria, IMI, Enterobacter cloacae complex, ECC, epidemiology, antimicrobial drug resistance, France, Mayotte, La Réunion

## Abstract

We characterized a collection of IMI-like–producing *Enterobacter* spp. isolates (n = 112) in France. The main clone corresponded to IMI-1–producing sequence type 820 *E. cloacae* subspecies *cloacae* that was involved in an outbreak. Clinicians should be aware of potential antimicrobial resistance among these bacteria.

The *Enterobacter cloacae* complex (ECC) is highly diverse; its many species and subspecies can be distinguished by using phenotypic methods or matrix-assisted laser desorption/ionization time-of-flight mass spectrometry. Whole-genome sequencing enables the precise determination of the bacterial species inside this complex; 22 species, including 6 subspecies, have been assigned to the ECC. IMI and NmcA, which are Ambler class A carbapenemases conferring antimicrobial resistance, are typically associated with the ECC (*1*), but they are rarely reported in other bacterial species (*2*,*3*) despite a worldwide distribution.

A total of 24 NmcA/IMI-type variants have been identified in accordance with the Beta-Lactamase DataBase (http://www.bldb.eu) (*4*). The *bla*_IMI/NmcA_ genes can be either chromosome or plasmid encoded; *bla*_NmcA_, *bla*_IMI-1_, *bla*_IMI-4_ and *bla*_IMI-9_ have been described as chromosome encoded (*5*–*7*). The chromosome-encoded *bla*_IMI/NmcA_ genes are usually described into XerC/XerD recombinase-dependent integrative mobile elements (IMEX) called *Eclo*IMEX elements. For all IMI producers, the genetic features showed an integration of *Eclo*IMEX structures at the same position between *setB* and *yeiP* genes. For chromosomal variant, the *bla*_IMI_ gene were mostly identified in *E. cloacae* subsp. *cloacae* as *E. bugandensis* or *E. ludwigii* strains (*6**,**8**,**9*). In contrast, the plasmid-encoded genes (such as *bla*_IMI-2_ or *bla*_IMI-6_) were mostly identified on a IncFII(Yp)-type plasmid in *E. asburiae* isolates (*3*,*6*,*10*). We characterized a large collection of IMI/NmcA producers collected in France.

## The Study

We included all nonduplicate IMI-producing and NmcA-producing isolates showing antimicrobial resistance received at the French National Reference Center for Antimicrobial resistance (F-NRC) during 2012–2022 (n = 112) (Appendix 1 Table 1). Mass spectrometry showed that all strains belonged to the ECC. Since 2014, each year, 3–20 IMI/NmcA producers were identified, representing 0.03%–0.91% of all carbapenemase-producing Enterobacterales analyzed at F-NRC. No IMI/NmcA producers were found before 2014. (Appendix 2 Figure 1). 

Disc diffusion antimicrobial susceptibility testing revealed resistance to third-generation cephalosporins for 1 strain (257D9, overexpression of *ampC* confirmed with CLOXA agar) of the 112 tested. We determined MICs for last-resort antibiotics against highly resistant bacteria on 30 IMI/NmcA producers belonging to several sequence types (STs) (Appendix 1 Table 2). Relebactam restored imipenem activity for 67% of the strains and vaborbactam restored susceptibility to meropenem for all strains with lower MICs than imipenem/relebactam. Then, 37% of the tested strains were susceptible to colistin. All 30 IMI/NmcA producers remained susceptible to cefepime, cefiderocol, and ceftazidime/avibactam. 

We performed WGS on the 112 IMI/NmcA producers and identified 74 IMI-1 producers (Appendix 2 Figures 1, 2). Of those, 44 IMI-1–producing ECC were involved in an outbreak in Mayotte and La Réunion islands.

We confirmed ECC species identification using average nucleotide identity (ANI) calculation (Appendix 1 Table 3; Appendix 3). *E. cloacae* subsp. *cloacae* was the most prevalent species (n = 56 [50.0%]) (Figure). Multilocus sequence typing (MLST) assigned 42 known unique STs for 105 strains. The 7 remaining isolates belonged to new or undetermined STs. Major STs (>4 isolates) were ST820 (n = 45), ST250 (n = 5), ST657 (n = 5), ST1516 (n = 4), and ST1517 (n = 4) (Figure). Of note, 44 of the ST820 strains corresponded to the strain isolated in the Mayotte/La Réunion outbreak; the last IMI-1 *E. cloacae* subsp. *cloacae* of ST820, 193I8, was isolated in Paris and was not clonally related to the outbreak strains. That strain exposed >1,200 single-nucleotide polymorphisms (SNPs) corresponding with the other IMI-1 ECC ST820 isolates from Mayotte or La Réunion.

**Figure:**
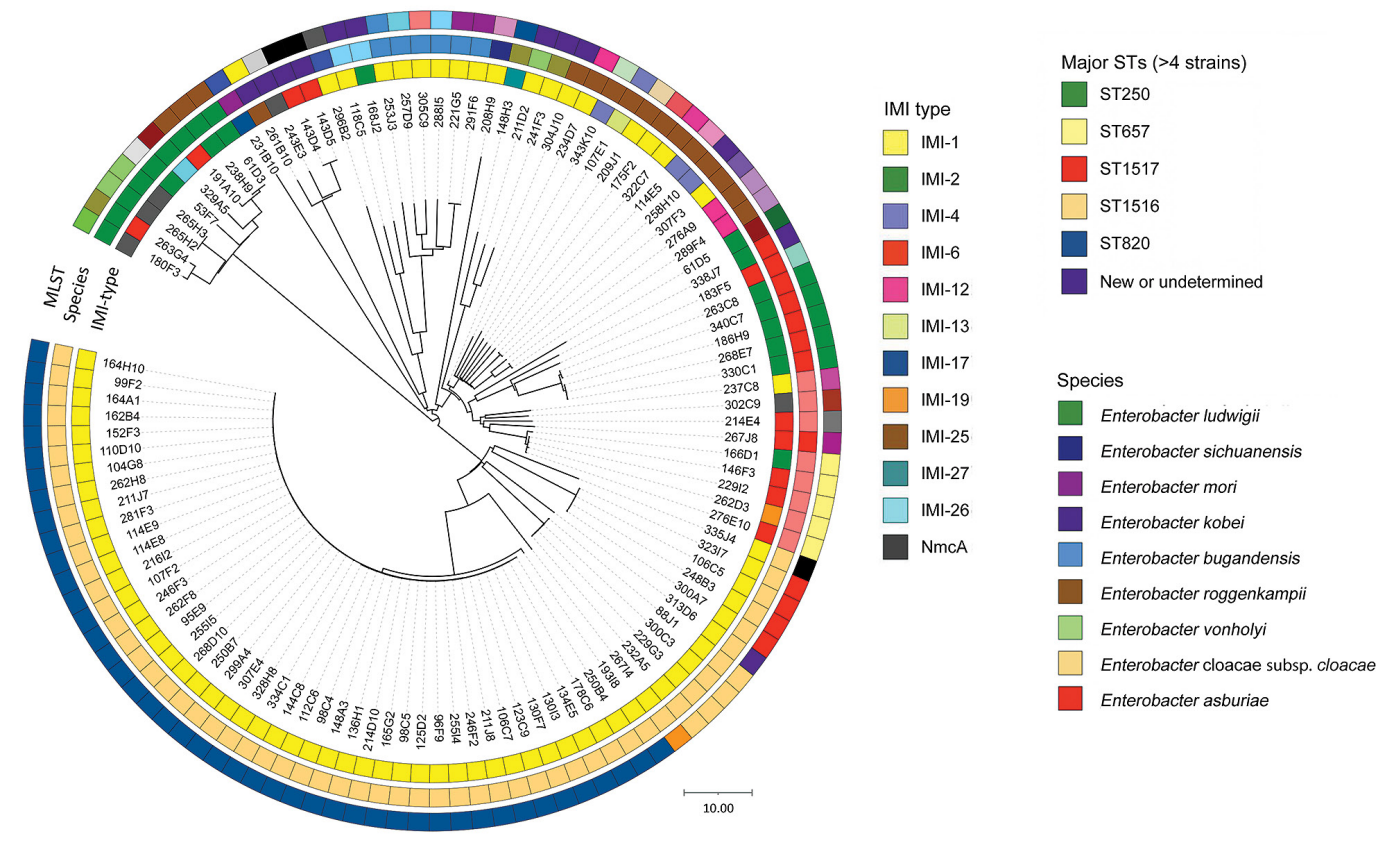
Phylogenetic relationship and global characterization of 112 IMI-producing *E. cloacae* complex received by the French National Reference Center, France, 2012–2022. The phylogenetic tree was built with a single-nucleotide polymorphism analysis approach from whole-genome sequencing data. MLST, multilocus sequence type; ST, sequence type.

Genes encoding NmcA, IMI-1, IMI-4, IMI-12, and IMI-13 were localized on the chromosome, whereas those coding for IMI-2, IMI-6, IMI-17, IMI-19, IMI-25, IMI-26 and IMI-27 were carried on plasmids. We characterized genetic environments of *bla*_IMI/NmcA_ genes using Illumina (https://illumina.com) and MinION long-read (Oxford Nanopore, https://nanoporetech.com) sequencing. All chromosome-encoded *bla*_IMI/NmcA_ genes were located into a *Eclo*IMEX-type genetic element (Appendix 2 Figure 4, panel A), except *bla*_IMI-13_, which possessed a distinct genetic environment (Appendix 2 Figure 4, panel B). We detected already-characterized *Eclo*IMEX-type and 6 new variants, named *Eclo*IMEX-11−16 (Appendix 2 Figure 4, panel A). Those *Eclo*IMEX elements were ≈15–≈39.4-kb long, possessed a highly conserved 5′ region, and were inserted between *setB* and *yieP* genes. We observed a strong correlation between *bla*_NmcA_ and *Eclo*IMEX-1. In contrast, we identified *bla*_IMI-1_ on 9 different *Eclo*IMEX elements. We saw no correlation between the *Enterobacter* species and the type of *Eclo*IMEX. The *bla*_IMI-13_ gene was inserted in the chromosome between genes encoding a hypothetical protein and an Inovirus-type Gp2 protein. We identified several complete or partially deleted insertion sequences (IS) close to *bla*_IMI-13_ (Appendix 2 Figure 4, panel B); however, the mechanism of *bla*_IMI-13_ acquisition is unclear.

All *bla*_IMI-6_ genes were carried on a IncFII(Yb)-type plasmid (160–200 kb) (Appendix 1 Table 4). Similarly, *bla*_IMI-2_ genes were carried on a IncFII(Yp)-type plasmid for 75% (8/12) of the IMI-2 producers. The plasmidic replicase was not identified in the 4 remaining IMI-2 producers. The long-read sequencing performed on strains producing new IMI variants enabled a more precise identification of plasmid type and size (Appendix 1 Table 3). The close genetic environments of the *bla*_IMI_ genes included several IS that differed according to the *bla*_IMI_ variants (Appendix 2 Figure 4). Conjugation experiments performed in *E. coli* J53 used as recipient strain confirmed those plasmids were conjugative except the 1 carrying *bla*_IMI-17_.

We built an SNP matrix for the 44 IMI-1 *E. cloacae* subsp. *cloacae* ST820 isolates involved in the Mayotte/La Réunion outbreak to confirm their clonality. Those strains were closely related (1–62 SNPs between 2 isolates). We also performed a Bayesian analysis to estimate the date of the most recent ancestor and the evolutionary rate of that population. We estimated the evolutionary rate of the clone to 3.94 × 10^−7^ substitutions per site and per year (95% highest posterior density [HPD] 2.50–5.33 × 10^−7^), corresponding to 1.63 SNPs per genome per year (95% HPD 1.04–2.21 SNPs). The common ancestor of the 44 IMI-1–producing *E. cloacae* subsp. *cloacae* ST820 isolates has an estimated date of 1994.7 (95% HPD 1990.8–2000.2) (Appendix 2 Figure 5).

## Conclusions

Consistent with previous findings (*6*,*9*), our collection of IMI producers included uncommon species of ECC, such as *E. cloacae* subsp. *cloacae*, a rarely described species; IMI-1, IMI-2 and IMI-6 were the most prevalent variants. We identified no isolates of *E. hormaechei*, the most prevalent carbapenemase-producing ECC species (*11*,*12*). 

Genetic environments and plasmid types of IMI-2 producers identified in this study were similar to those previously described (*2*,*3*,*13*); IncFII(Yp)-type plasmids were most common. The close genetic environment of *bla*_IMI-2_ observed in our isolates has been reported on a plasmid identified in *E. coli* (*2*). The genetic environment of *bla*_IMI-6_ was previously reported in an *E. cloacae* isolate described by Boyd et al. (*6*). Regarding the chromosome-encoded IMI and NmcA variants (n = 85), we described a variety of *Eclo*IMEX elements (n = 11) including 6 novel elements; that the same *Eclo*IMEX could be identified in different ECC species suggests that XerC/D recombinases enable the mobility of these *bla*_IMI-/NmcA_–carrying *Eclo*IMEX structures specifically between ECC species. Finally, the evolution rate of the IMI-1–producing *E. cloacae* subsp. *cloacae* ST820 clone (1.63 SNPs/genome/year) is similar to the 0.5–3 SNPs/year for a genome reported for a population of multidrug-resistant ECC in the United Kingdom (*14*) and the 2.5–3 SNPs/year for a genome identified for ST171 and ST78 carbapenem-resistant ECC (*15*).

In conclusion, in IMI/NmcA producers in France, we observed a large diversity of ECC species, STs, genetic supports, and genetic environments. Future work should elucidate why *E. cloacae* subsp. *cloacae* is highly prevalent among IMI producers; why *bla*_IMI/NmcA_-carrying plasmids were almost always found alone in IMI-producing isolates that always do not carry any other resistance genes; and whether *Eclo*IMEX genetic elements are mobilizable. Clinicians should remain aware of potential antimicrobial resistance among ECC species.

Appendix 1 Additional tables for IMI-type carbapenemase-producing *E. cloacae* complex, France and overseas regions, 2012–2022.

Appendix 2 Additional information about IMI-type carbapenemase-producing *E. cloacae* complex, France and overseas regions, 2012–2022.

Appendix 3 Additional calculations for IMI-type carbapenemase-producing *E. cloacae* complex, France and overseas regions, 2012–2022.
