# Supplementary material for: IMI-Type Carbapenemase-Producing Enterobacter cloacae Complex, France and Overseas Regions, 2012–2022
Source: Emerg Infect Dis. 2024 Jun;30(6):1279–82. doi: 10.3201/eid3006.231525 (PMC11138976; doi:10.3201/eid3006.231525)
Supplement: Appendix 2 — Additional information about IMI-type carbapenemase-producing E. cloacae complex, France and overseas regions, 2012–2022. [file 23-1525-Techapp-s2.pdf]

Article DOI: http://doi.org/10.3201/eid3006.231525

EID cannot ensure accessibility for supplementary materials supplied by authors. Readers who have difficulty accessing supplementary content should contact the authors for assistance.

IMI-type Carbapenemase-Producing *Enterobacter cloacae* complex, France and Overseas Regions, 2012–2022

Appendix

Material and Methods

Strains

All non-duplicate IMI- or NmcA-producing isolates received at the French National Reference Center (F-NRC) for antimicrobial resistance from 2012 to 2022 were included in this study (n=112). Theses isolates were from France including overseas French territories. Among them, 44 IMI-1-producing ECC were involved in an outbreak in Mayotte and La Réunion islands (Indian Ocean). This outbreak has been ongoing since 2015 on these neighbouring islands.

Overall, these clinical isolates were isolated from rectal swabs (n=99), urine (n=4), deep surgical sites (n=2), blood culture (n=1), a respiratory tract sample (n=1), or of unknown origin (n=5). Identification using MALDI-TOF-MS (Bruker Daltonics) showed that all strains belonged to the *Enterobacter cloacae* complex (ECC). Appendix 1 Table 1 recapitulates (https://wwwnc.cdc.gov/EID/article/30/6/23-1525-App1.xlsx) the characteristics of these 112 strains.

Antimicrobial Susceptibility Testing

Antimicrobial susceptibility testing was performed by disc diffusion method for all 112 IMI/NMC-A producers according to EUCAST recommendations. Minimal Inhibitory Concentrations (MICs, mg/L) to imipenem, imipenem-relebactam, meropenem, meropenem-vaborbactam, cefiderocol, ceftolozane-tazobactam, ceftazidime-avibactam, piperacillin-tazobactam, aztreonam, cefepime and colistin were performed by broth microdilution (UMIC®

for cefiderocol and EUMDROXF Sensititre™ for the other antibiotics) for 30 representative IMI-producers of different STs (Appendix 1 Table 2).

Whole-Genome Sequencing (WGS) and Phylogenetic Analysis

A short-read next-generation sequencing (WGS) was performed on all 112 IMI-like-producing ECC isolates using the HiSeq system (Illumina, San Diego, CA; GenBank accession number: PRJNA1015552). Illumina reads were assembled using Shovill v1.1.0 and spades v3.14.0. MLST and resistome analysis were performed using pubMLST and Resfinder databases. ECC species identification was confirmed with WGS data using Centrifuge Taxonomic Classifier 1.0.3 (https://github.com/chienchi/kbase-centrifuge). For phylogenetic analysis, WGS sequence reads were mapped to the reference genome (*E. cloacae* complex 96F9) using SNIppy v4.6.0, trees were built using PhyloTree and a SNPs matrix was built for the 44 IMI-1-producing ECC involved in the Mayotte/La Réunion islands outbreak. Phylogenetic trees were visualized using iTOL v6.5.2. Genetic environments were characterized by studying contigs carrying the $bla_{\text{IMI/NmcA}}$ genes and by mapping Illumina reads to different characterized *Eclo*IMEX-type elements using CLC Genomic Workbench v21.0.5 software (Qiagen, Les Ulis, France). Integrons were classified according to INTEGRALL (http://integrall.bio.ua.pt/). Long-read sequencing was performed for all strains with an undetermined genetic background (n=16) and for ECC producing novel and undescribed IMI-type carbapenemases (n=7) using Oxford nanopore MinION technology (Oxford Nanopore, Oxford, United-Kingdom) as previously described (*1*). By combining data obtained by Illumina and MinION sequencing, we reconstructed the different plasmids and genetic environments using the software CLC Genomics Workbench v21.0.5.

Transferability of the bla_{IMI} Genes and Plasmids Characterization

We performed a conjugation experiment to evaluate the transferability of the bla_{IMI} genes to other bacterial strains for ECC producing novel and undescribed plasmid-encoded IMI-type carbapenemases. We conducted this conjugation assay using the azide-resistant *E. coli* J53 as the recipient in Luria–Bertani broth using a 1:4 donor-to-recipient ratio. Transconjugants were selected on agar plates supplemented with imipenem (1 µg/mL) and azide (100 µg/mL).

Due to various insertion sequencing (IS) surrounding the bla_{IMI} genes, Illumina sequencing doesn’t allow replicase identification of plasmids carrying these genes. Accordingly,

we performed long-read sequencing only for new plasmid-encoded variants (IMI-27, IMI-25) and those that have never been described in the literature (IMI-17, IMI-19 and IMI-26). Plasmid contents of clinical isolates were identified by searching for replicase genes using the PlasmidFinder pipeline.

For IMI-2 and IMI-6 producing isolates, the plasmids were transferred by conjugation, the bla_{IMI-2} and bla_{IMI-6} -carrying plasmids were characterized according to their incompatibility group, using the PCR-based typing method designed by Carattoli *et al* (*2*). For these strains, the sizes of bla_{IMI}-carrying plasmids were determined using the Kieser extraction method (*3*).

Molecular Clock and Bayesian Phylogenetic Analysis of IMI-1-Producing *E. cloacae* subsp. *cloacae* Responsible for the Outbreak in Mayotte/La Réunion Islands

Using sequencing data obtained on the 44 IMI-1 *E. cloacae* subsp. *cloacae* ST820 involved in the Mayotte and La Réunion islands outbreak, we performed a Bayesian analysis to estimate the date of the most recent ancestor and the evolutionary rate of this population. We first investigated the temporal signal using IQTree v1.6.12 and TempEst v1.5.3 software (*4*,*5*). We then performed a Bayesian analysis using the BEAST v2.6.1 software. We used the core SNPs defined by SNIppy v4.6.0 as input, without exclusion of recombination regions. A GTR model was chosen as the best fit model using IQTree v1.6.12, and we tested both strict and relaxed log-normal molecular clock models. For each parameter set, we performed 100 million generations with sampling every 5,000 states. We used Tracer v1.7.2 to explore evolutionary rate and ensure an effective sample size (ESS) greater than 200. Additionally, we used TreeAnnnotator V1.10.4 and FigTree V1.4.4 to determine the age of the root nodes of the phylogenetic tree.

References

1. Girlich D, Bonnin RA, Proust A, Naas T, Dortet L. Undetectable production of the VIM-1 carbapenemase in an *Atlantibacter hermannii* clinical Isolate. Front Microbiol. 2021;12:741972. PubMed https://doi.org/10.3389/fmicb.2021.741972

2. Carattoli A, Bertini A, Villa L, Falbo V, Hopkins KL, Threlfall EJ. Identification of plasmids by PCR-based replicon typing. J Microbiol Methods. 2005;63:219–28. PubMed https://doi.org/10.1016/j.mimet.2005.03.018

3. Kieser T. Factors affecting the isolation of CCC DNA from *Streptomyces lividans* and *Escherichia coli*. Plasmid. 1984;12:19–36. PubMed https://doi.org/10.1016/0147-619X(84)90063-5

4. Rambaut A, Lam TT, Max Carvalho L, Pybus OG. Exploring the temporal structure of heterochronous sequences using TempEst (formerly Path-O-Gen). Virus Evol. 2016;2:vew007. PubMed https://doi.org/10.1093/ve/vew007

5. Nguyen LT, Schmidt HA, von Haeseler A, Minh BQ. IQ-TREE: a fast and effective stochastic algorithm for estimating maximum-likelihood phylogenies. Mol Biol Evol. 2015;32:268–74. PubMed https://doi.org/10.1093/molbev/msu300

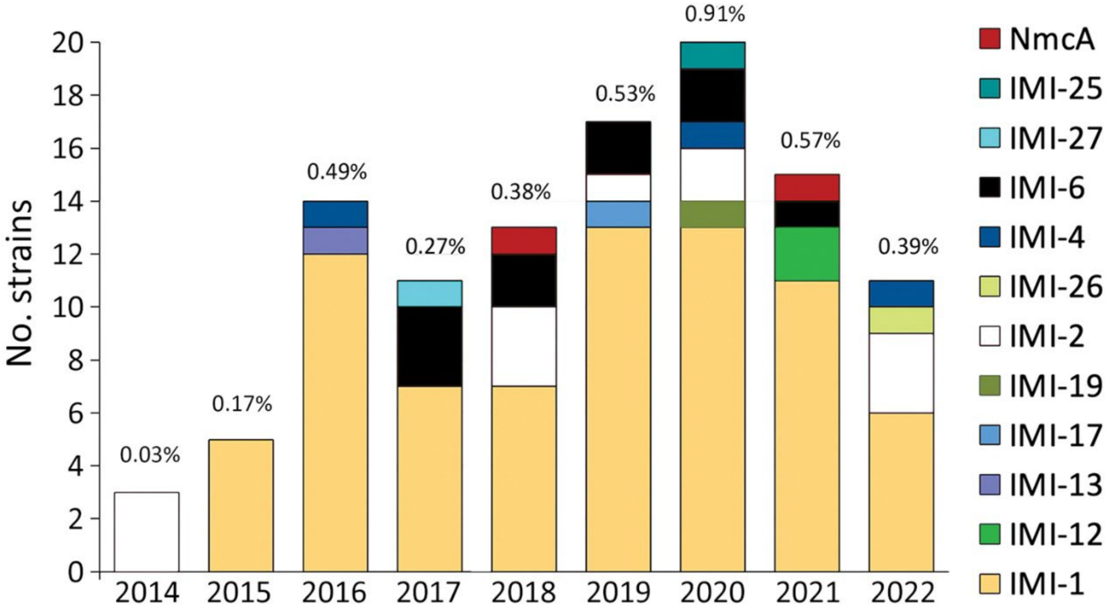

Appendix 2 Figure 1. Description of IMI-producing *Enterobacterales* received at the French National Reference Center for Carbapenem-resistant *Enterobacterales*, 2012–2022. No IMI/NmcA producers were reported before 2014. The percentage in red corresponds to the proportion of IMI-type–producing carbapenem-resistant *Enterobacterales* spp. among all CPE received at the reference center each year. CPE, carbapenem-resistant Enterobacterales.

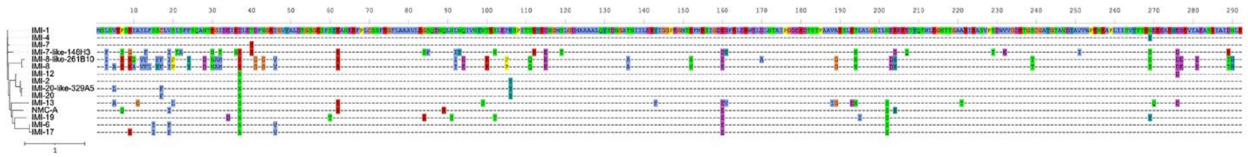

Appendix 2 Figure 2. Protein alignment of the different IMI/NMC-A variants included in this study.

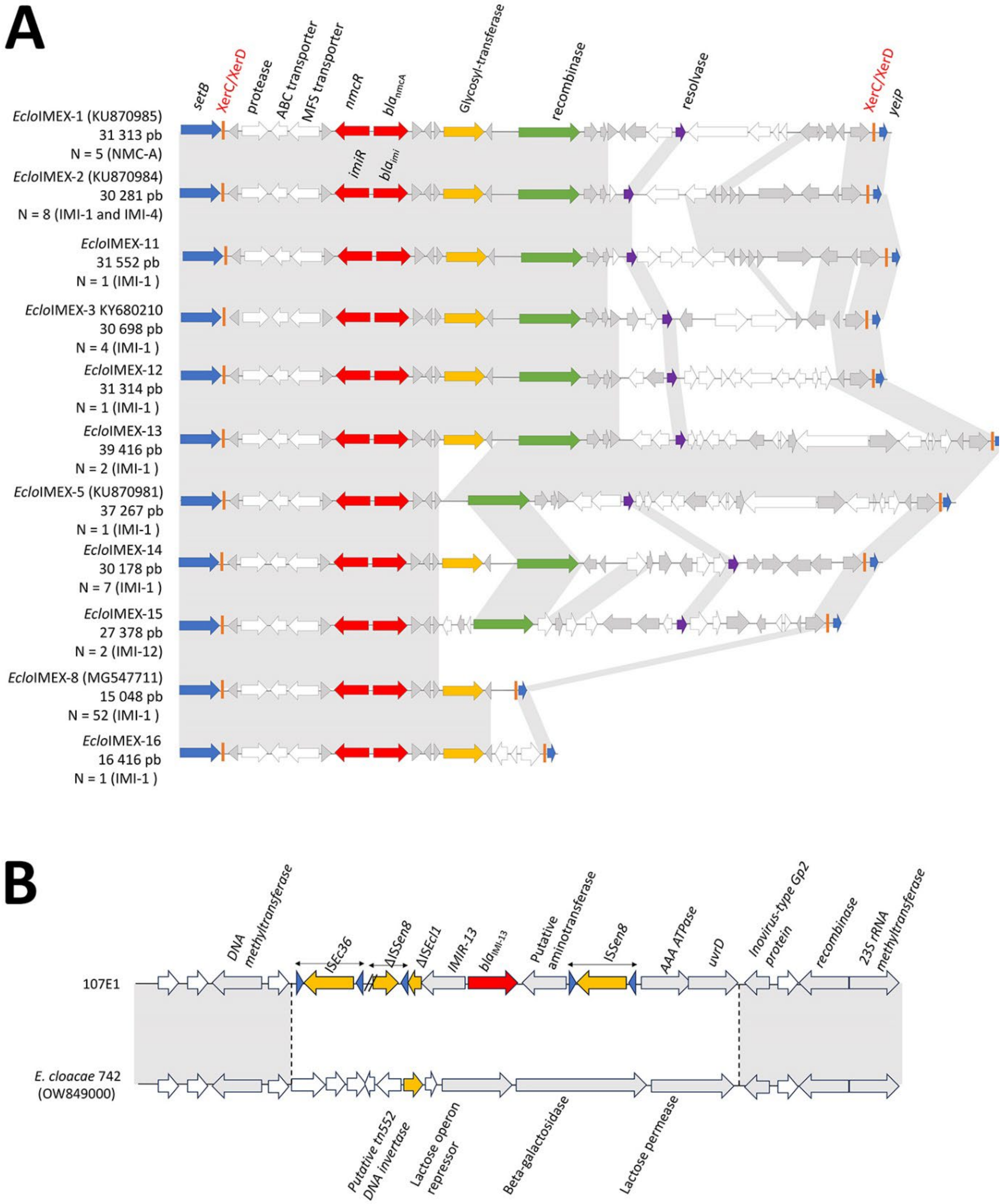

Appendix 2 Figure 3. Close genetic environments of chromosome-encoded blaIMI/NmcA genes (in color and gray: genes encoding proteins with known functions, in white: genes encoding hypothetical proteins). A) XerC/D recombinase-dependent integrative mobile elements and B) non XerC/D recombinase-dependent integrative mobile elements.

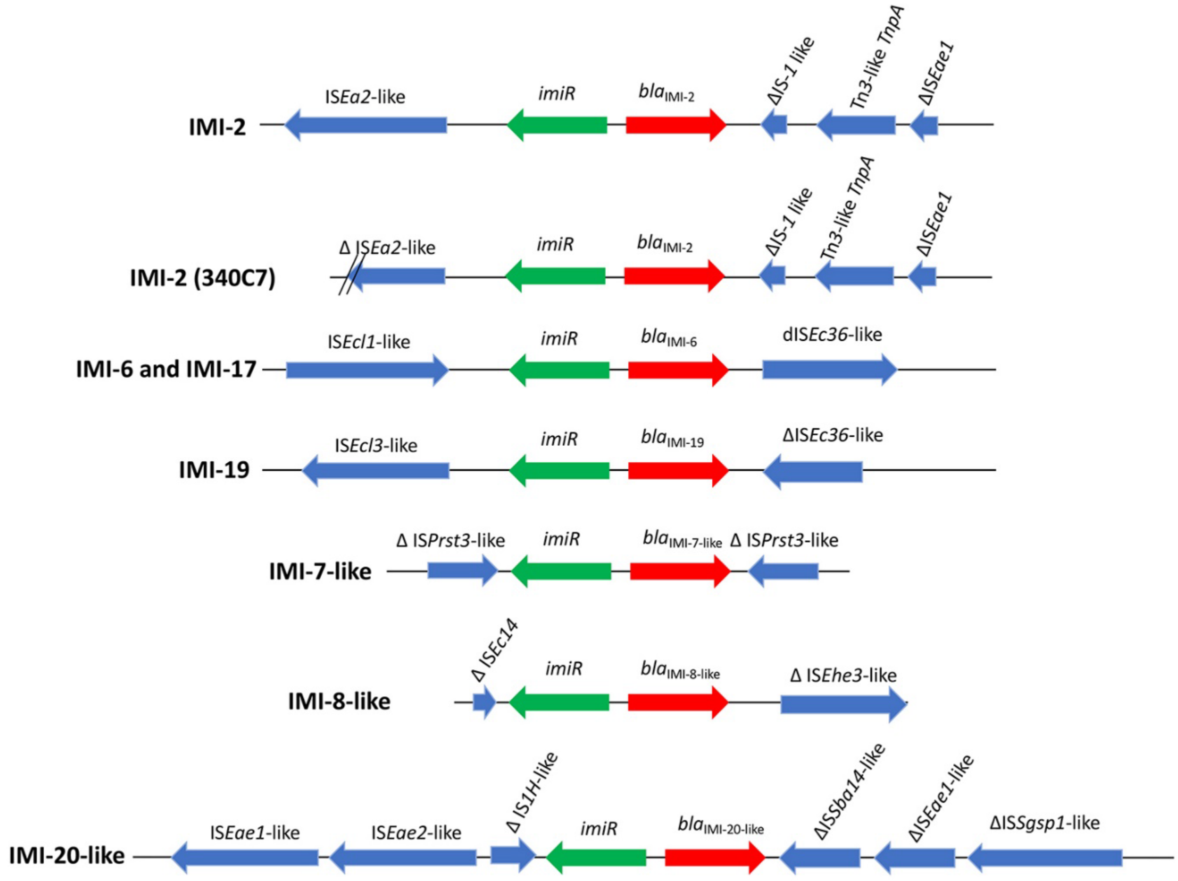

Appendix 2 Figure 4. Close genetic environments of plasmid-encoded blaIMI genes included in this study.

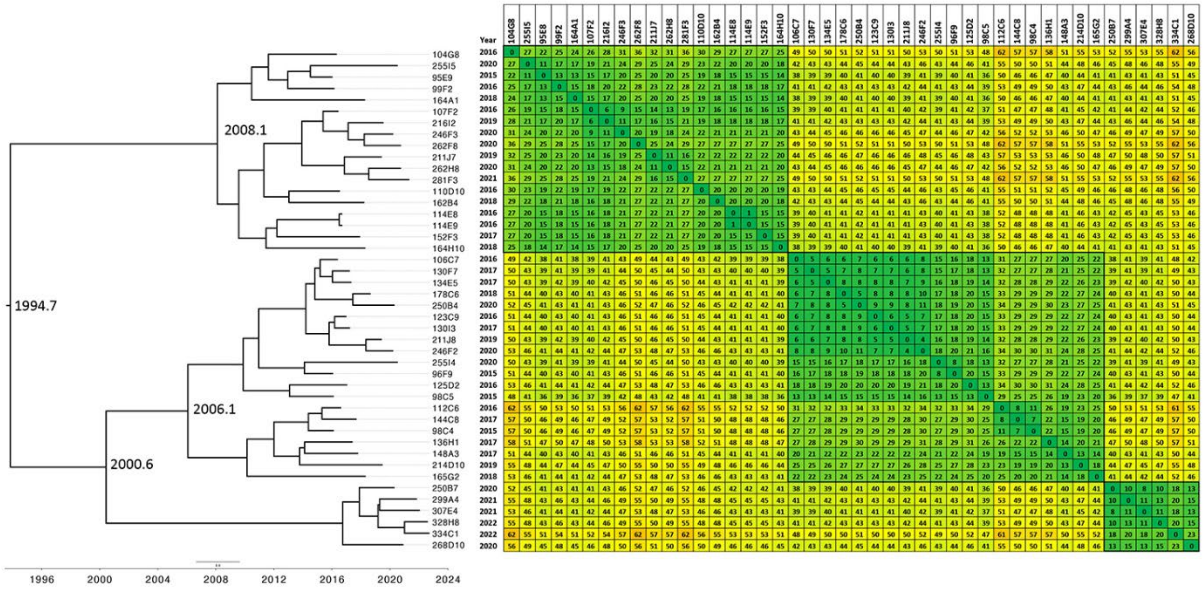

Appendix 2 Figure 5. Time-scaled Bayesian phylogeny and SNPs matrix for the 44 IMI-1 producing *E. cloacae* subsp. cloacae ST820 involved in the outbreak in Mayotte and La Réunion Islands.
